# Supplementary material for: The Ps and Qs of alarmone synthesis in Staphylococcus aureus
Source: PLoS One. 2019 Oct 15;14(10):e0213630. doi: 10.1371/journal.pone.0213630 (PMC6793942; doi:10.1371/journal.pone.0213630)
Supplement: S1 Table — (PDF) [file pone.0213630.s001.pdf]

S1 Table. Sequences of PCR Primers Used

Primer name	Primer sequence
M13 Forward	GTAAAACGACGGCCAG
M13 Reverse	CAGGAAACAGCTATGAC
Sa-rel-Fb2	CGCGGATCCATGAACAACGAATATCCATATAGTG
Sa-rel-Re2	CCGGAATTCCTAGTTCCAAACTCTTGTTACTGTATA
Sa-rel-tFnco	CATGCCATGGGCAACAACGAATATCCATATAGTGC
Sa-rel-tRsal	ACGCGTCGACTCAGTGGTGGTGGTGGTGGCTGCTTCCTCC GGTTTCCATAAATTCTTGAGCG
SARelPfor	ATATGGATCCATGTATGTAGATCGAAAACCATCAC
SAVRelPrev	ATATCTCGAGCTACTCTGTTATTTCAGAATGAATTTG
SARelQfor	ATATGGATCCCATATGAATCAATGGGATCAGTTCTTAAC
SARelQrev	AATTCTCGAGTTAAAGCTTATCATTTTCATGTTTTTTAGAACGTTT
SA1919for1	ATATGGATCCCATATGGCTAAAACATATATTTTCGGACA
SA1919rev1	TTAACTCGAGTTAAAGCTTTTTTGTTAATGCTTCAGTGATTT
SMRelPfor	ATATGGATCCATGTCACAAGAAACGATCTATGG
SMRelPrev	ATATCTCGAGTTATTCACCACTTCCTACAATC
SMRelQfor	ATATGGATCCATGAATTGGGAAGAATTTCTGG
SMRelQrev	ATATCTCGAGTTATCGGTAAAGTTCATCTGTG
