# Supplementary material for: The Ps and Qs of alarmone synthesis in Staphylococcus aureus
Source: PLoS One. 2019 Oct 15;14(10):e0213630. doi: 10.1371/journal.pone.0213630 (PMC6793942; doi:10.1371/journal.pone.0213630)
Supplement: S2 Table — (PDF) [file pone.0213630.s002.pdf]

S2 Table. Sa-RelP and Sa-RelQ molar specific activities with/without ppGpp stimulation at pH 7.6

**Protein tested**	**Reaction**	**Reaction pH**	**Reaction rate with no alarmone added (µmol.min^{-1}.µmol^{-1})**	**Reaction rate with 100 µM ppGpp added (µmol.min^{-1}.µmol^{-1})**	**Fold change**
**Sa-RelQ**	GMP + ATP	pH 7.6	0.28 ± 0.04	9.64 ± 0.52	34 ± 1.9
**Sa-RelQ**	GMP + ATP	pH 9.0	0.46 ± 0.25	11.7 ± 0.8	26 ± 2
**Sa-RelQ**	GDP + ATP	pH 7.6	6.0 ± 0.84	9.44 ± 1.48	1.6 ± 0.3
**Sa-RelQ**	GDP + ATP	pH 9.0	15.0 ± 4.3	23.4 ± 3.1	1.6 ± 0.1
**Sa-RelQ**	GTP + ATP	pH 7.6	3.44 ± 3.40	4.96 ± 0.32	1.4 ± 0.2
**Sa-RelQ**	GTP + ATP	pH 9.0	8.25 ± 1.53	14.2 ± 3.1	1.7 ± 0.4
**Sa-RelP**	GMP + ATP	pH 7.6	3.0 ± 0.88	N.D.	-
**Sa-RelP**	GMP + ATP	pH 9.0	3.69 ± 0.47	2.65 ± 0.23	0.7 ± 0.1
**Sa-RelP**	GDP + ATP	pH 7.6	48.9 ± 2.3	N.D.	-
**Sa-RelP**	GDP + ATP	pH 9.0	99.1 ± 11.6	82.4 ± 8.0	0.8 ± 0.2
**Sa-RelP**	GTP + ATP	pH 7.6	18.0 ± 2.2	N.D.	-
**Sa-RelP**	GTP + ATP	pH 9.0	53.8 ± 18.0	45.5 ± 23.1	0.9 ± 0.5

This table summarizes the molar specific activities (i.e. reaction rates in units of micromoles of alarmone product formed per minute per micromole of protein) for Sa-RelQ and Sa-RelP proteins under standardized conditions at pH 7.6 in the absence of stimulatory alarmone (no added alarmone), as well as in the presence of 100 µM ppGpp. The values are reported as mean ± standard deviation, based on data from 2–4 experimental replicates. The fold-change represents the ratio of ppGpp-stimulated rate/non-stimulated rate, for each of the respective (pp)pGpp-synthesizing reactions. Data for reactions performed at pH 9.0 taken from Table 3.
