# Supplementary figures and images for: The Ps and Qs of alarmone synthesis in Staphylococcus aureus
Source: PLoS One. 2019 Oct 15;14(10):e0213630. doi: 10.1371/journal.pone.0213630 (PMC6793942; doi:10.1371/journal.pone.0213630)

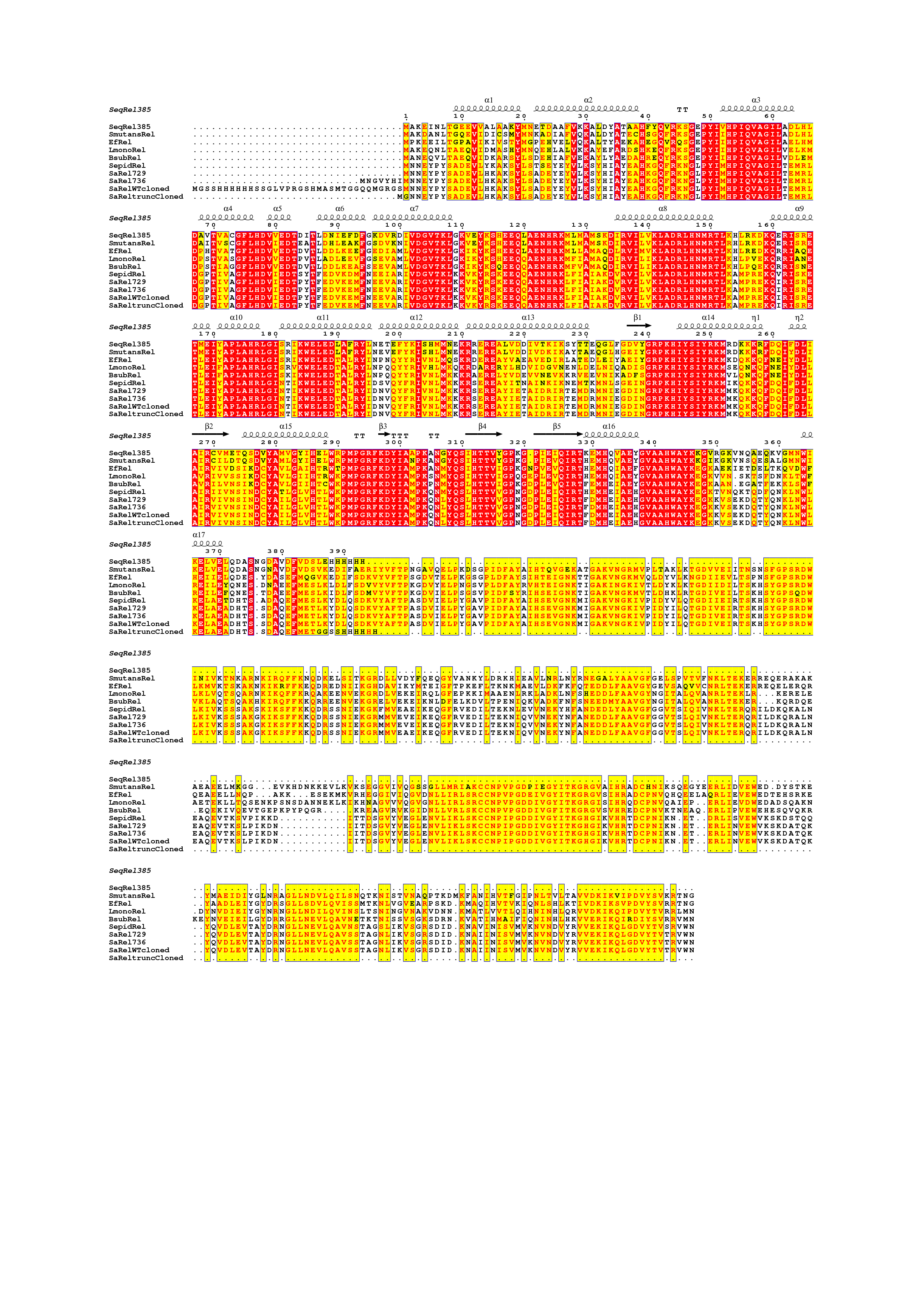

Supplement: S1 Fig — The multiple sequence alignment contains the amino acid sequences of the following long-Rel proteins: SeqRel385 (Streptococcus dysgalactiae subsp. equisimilis RelSeq NH 1–385; locus 1VJ7_A, 393 aa), SmutansRel (Streptococcus mutans UA159, locus tag SMU_2044, 740 aa); EFRel (Enterococcus faecalis V538, locus tag EF1974, 737 aa); LmonoRel (Listeria monocytogenes EDG-e, locus tag lmo1523, 738 aa); BsRel (Bacillus subtilis 168, locus tag BSU27600, 734 aa); SepidRel (Staphylococcus epidermidis ATCC 12228, locus tag SE_1315, 729 aa); SaRel729 (S. aureus Newman, locus tag NWMN_1536, residues 8–736); SaRel736 (S. aureus Newman, locus tag NWMN_1536, residues 1–736); SaRelWTcloned (recombinant Sa-Rel protein, this study, 763 aa); SaReltruncCloned (recombinant Sa-Reltrunc protein, this study, 396 aa). The aligned secondary structure elements for RelSeq NH 1–385 (PDB: 1VJ7) [23] are shown above the amino acid sequences. Numbering corresponds to the RelSeq NH 1–385 protein. See S1 Appendix for further details. (TIFF) [file pone.0213630.s003.tiff]

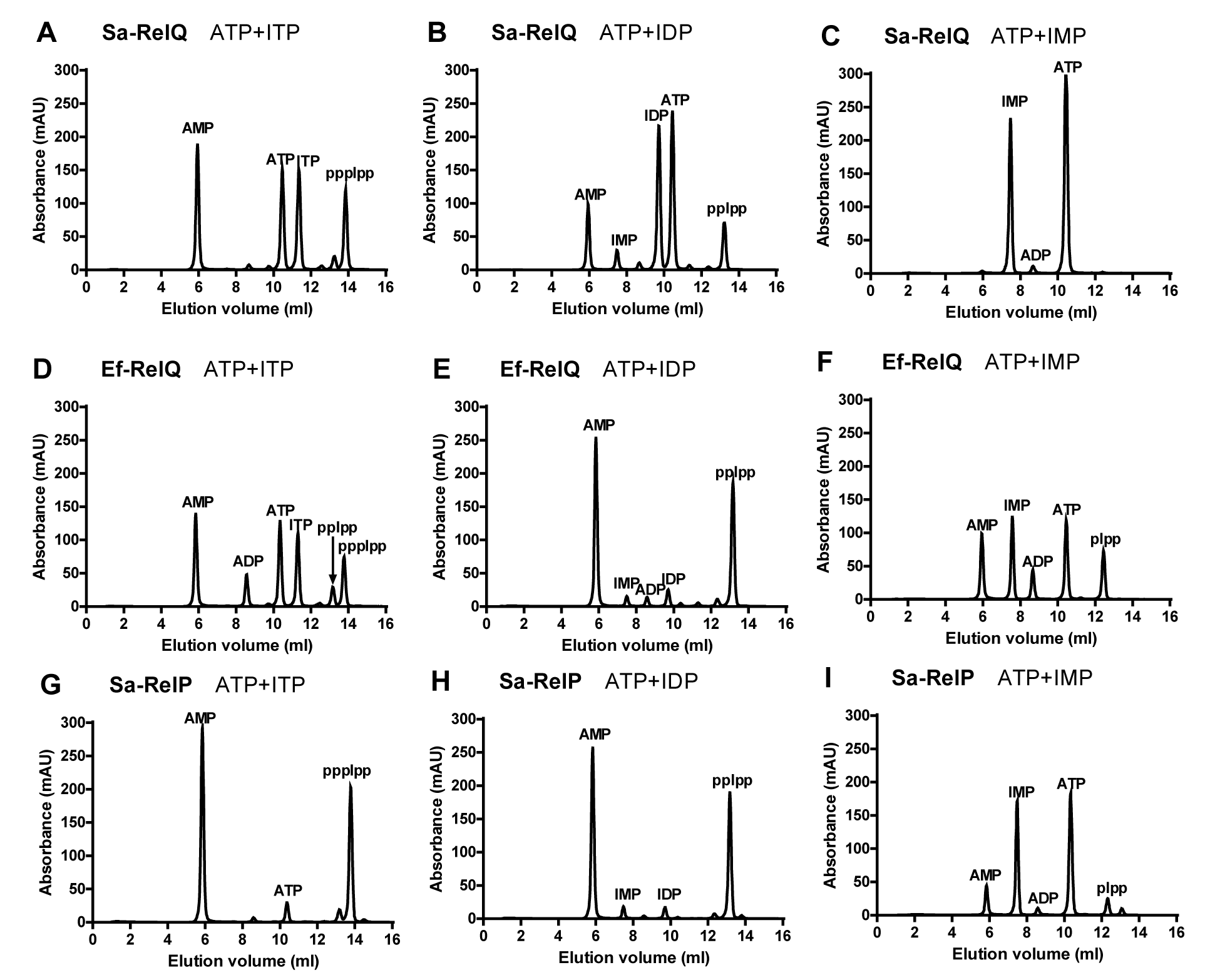

Supplement: S2 Fig — Panels A−I show representative anion-exchange chromatograms of product mixtures formed by the incubation of Sa-RelQ, Ef-RelQ and Sa-RelP with ATP+ ITP/IDP/IMP, under standardized conditions, to evaluate (pp)pIpp synthesis activities. (A) Sa-RelQ + ATP + ITP, (B) Sa-RelQ + ATP + IDP, (C) Sa-RelQ + ATP + IMP, (D) EF-RelQ + ATP + ITP, (E) EF-RelQ + ATP + IDP, (F) EF-RelQ + ATP + IMP, (G) Sa-RelP + ATP + ITP, (H) Sa-RelP + ATP + IDP, (I) Sa-RelP + ATP + IMP. The peaks corresponding to the respective substrates and products are indicated on each chromatogram. See Materials and methods and S1 Appendix for experimental details. (TIF) [file pone.0213630.s004.tif]

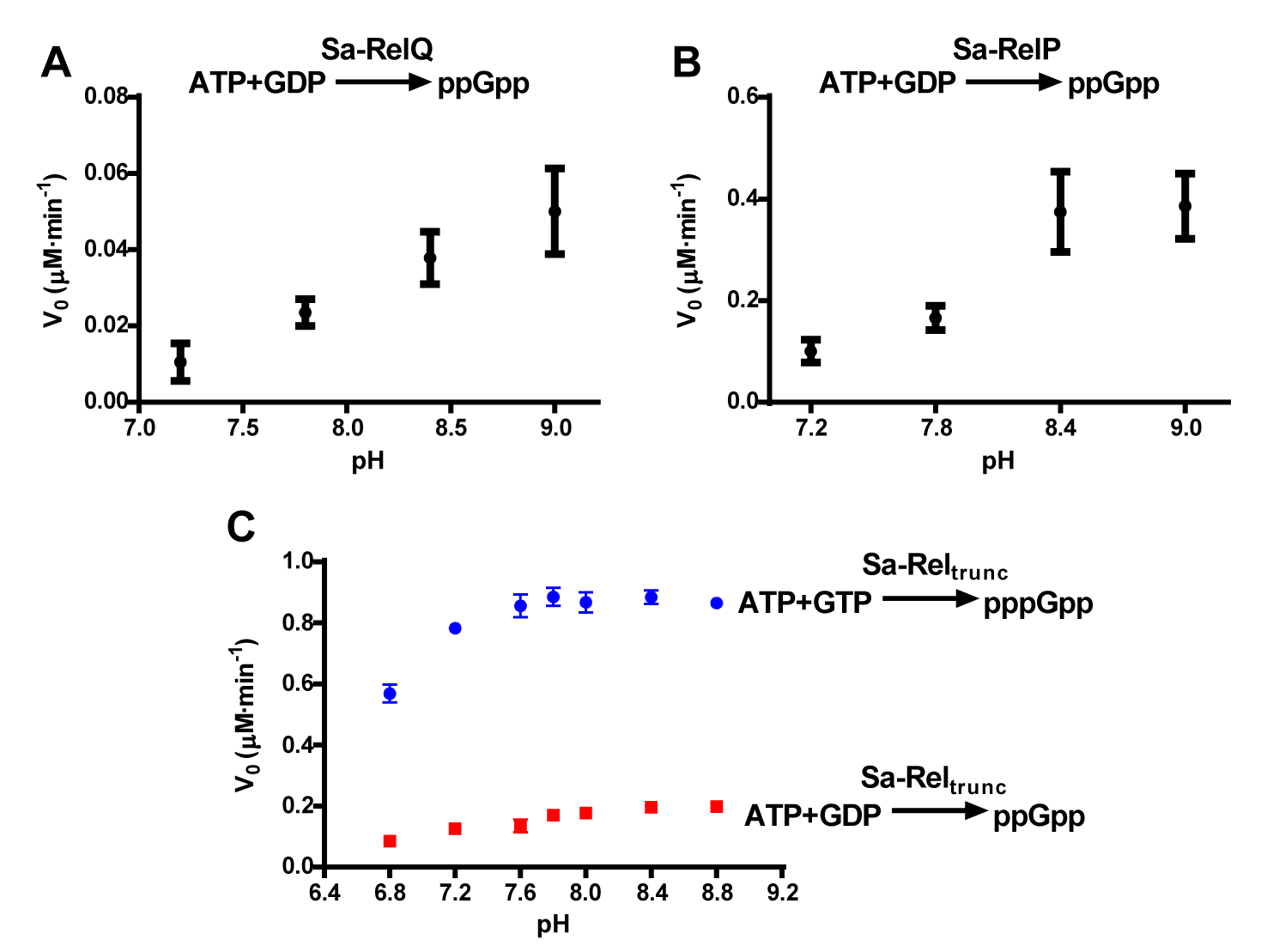

Supplement: S3 Fig — Panel A. Optimal pH for ppGpp synthesis by Sa-RelQ, determined over the range pH 7.2–9.0. Panel B. Optimal pH for ppGpp synthesis by Sa-RelP, determined over the range pH 7.2–9.0. Panel C. Optimal range for pppGpp (blue) and ppGpp (red) synthesis by Sa-Reltrunc, determined over the pH range 6.8 to 8.8. See Materials and methods and S1 Appendix for experimental details. (TIF) [file pone.0213630.s005.tif]

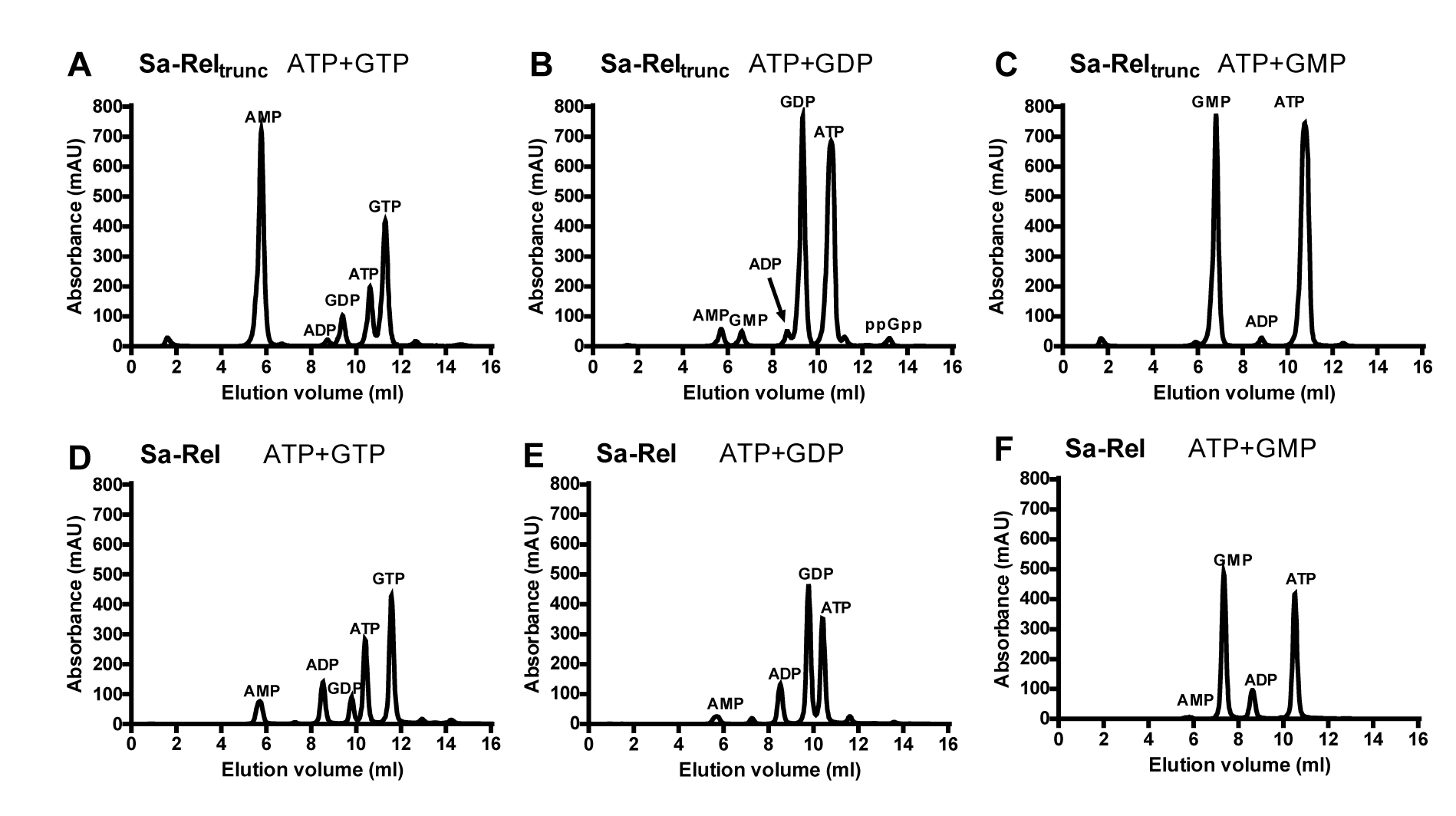

Supplement: S4 Fig — Panels A−F show representative anion-exchange chromatograms of product mixtures formed by the incubation of Sa-Rel and Sa-Reltrunc proteins with ATP+ GTP/GDP/GMP, under standardized conditions, to evaluate (pp)pGpp synthesis activities. (A) Sa-Reltrunc + ATP + GTP, (B) Sa-Reltrunc + ATP + GDP, (C) Sa-Reltrunc + ATP + GMP, (D) Sa-Rel + ATP + GTP, (E) Sa-Rel + ATP + GDP, (F) Sa-Rel + ATP + GMP. The peaks corresponding to the respective substrates and products are indicated on each chromatogram. See Materials and methods and S1 Appendix for experimental details. (TIF) [file pone.0213630.s006.tif]

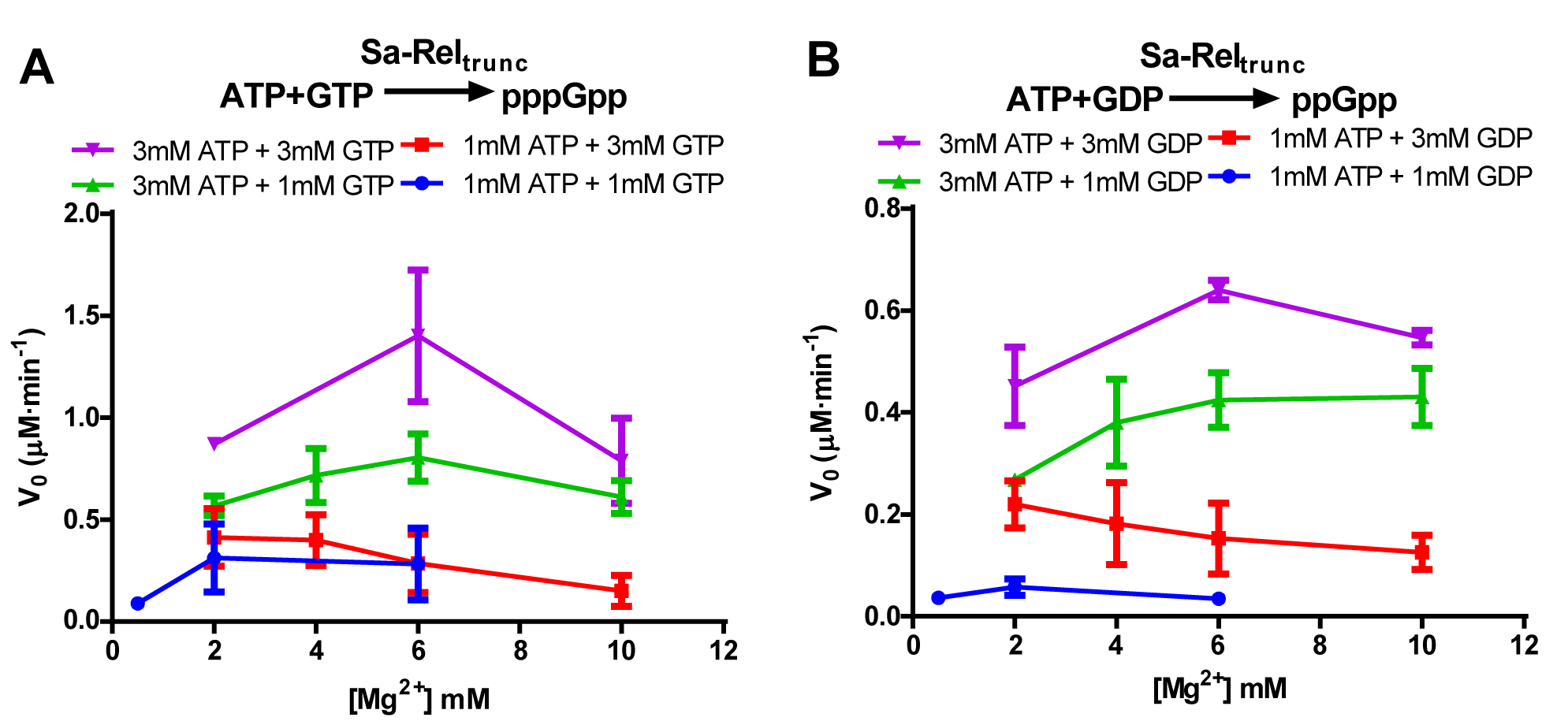

Supplement: S5 Fig — The rate of (p)ppGpp-synthesis by Sa-Reltrunc was determined under standardized conditions using 4 different molar ratios of ATP + GTP/GDP substrates, across a range of Mg2+ ion concentrations under standardized conditions, to evaluate their co-dependence. (A) Rate of pppGpp synthesis by Sa-Reltrunc. (B) Rate of ppGpp synthesis by Sa-Reltrunc. Conditions tested included: 3 mM ATP + 3 mM GTP/GDP, 3 mM ATP + 1 mM GTP/GDP, 1 mM ATP + 3 mM GTP/GDP, 1 mM ATP + 1 mM GTP/GDP, in the presence of 0.5 mM, 2 mM, 4 mM, 6 mM or 10 mM Mg2+ ions. The respective rates of (p)ppGpp synthesis (V0, in units of micromolar / min) are shown on Y-axis versus Mg2+ ion concentration. See Materials and methods and S1 Appendix for experimental details. (TIF) [file pone.0213630.s007.tif]

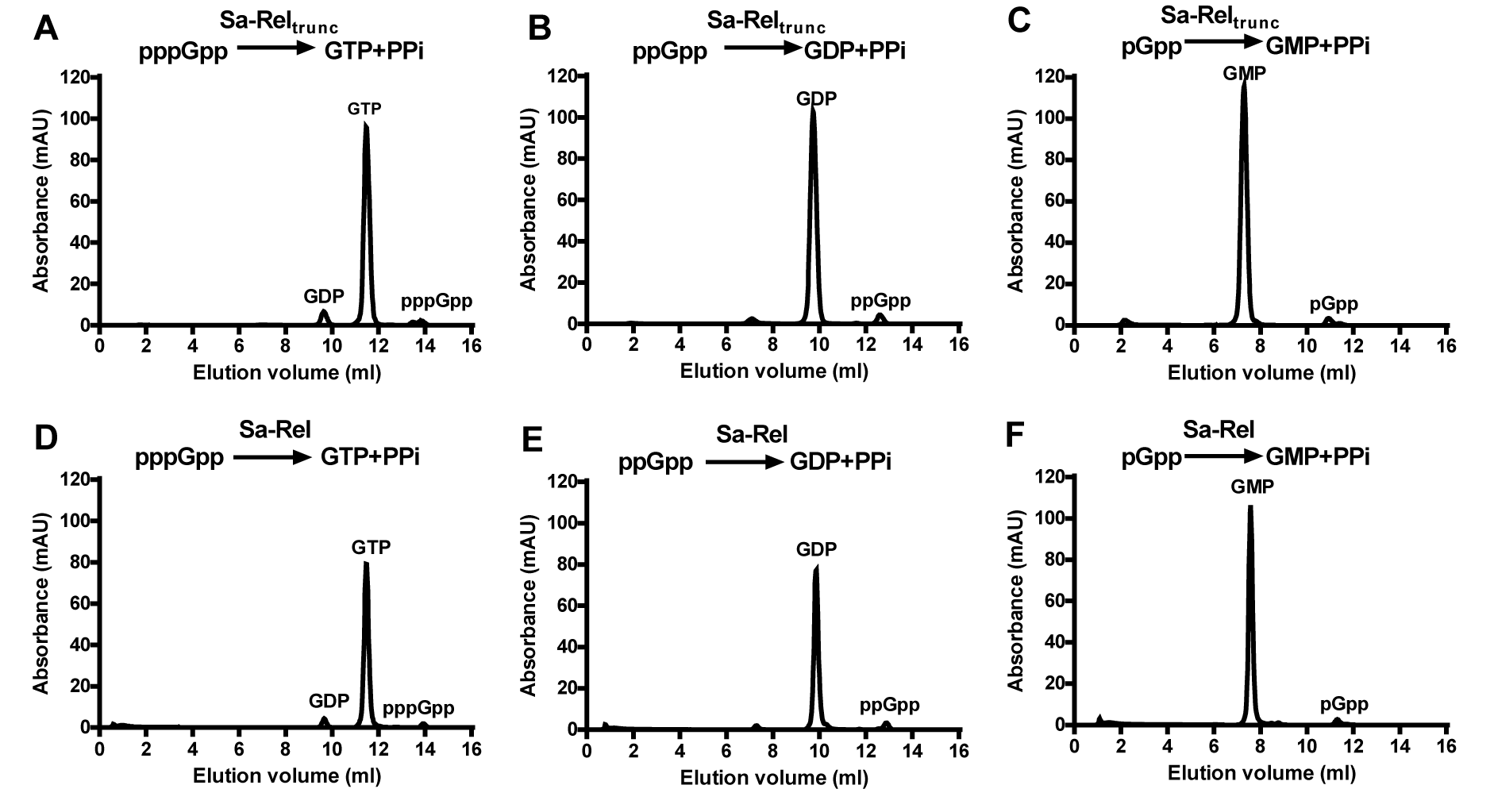

Supplement: S6 Fig — Panels A−F show representative anion-exchange chromatograms of product mixtures formed by the incubation of Sa-Rel and Sa-Reltrunc proteins with pppGpp, ppGpp or pGpp to evaluate hydrolytic activities. (A) Sa-Reltrunc + pppGpp, (B) Sa-Reltrunc + ppGpp, (C) Sa-Reltrunc + pGpp, (D) Sa-Rel + pppGpp, (E) Sa-Rel + ppGpp, (F) Sa-Rel + pGpp. The peaks corresponding to the respective products are indicated on each chromatogram. See Materials and methods and S1 Appendix for experimental details. (TIF) [file pone.0213630.s008.tif]

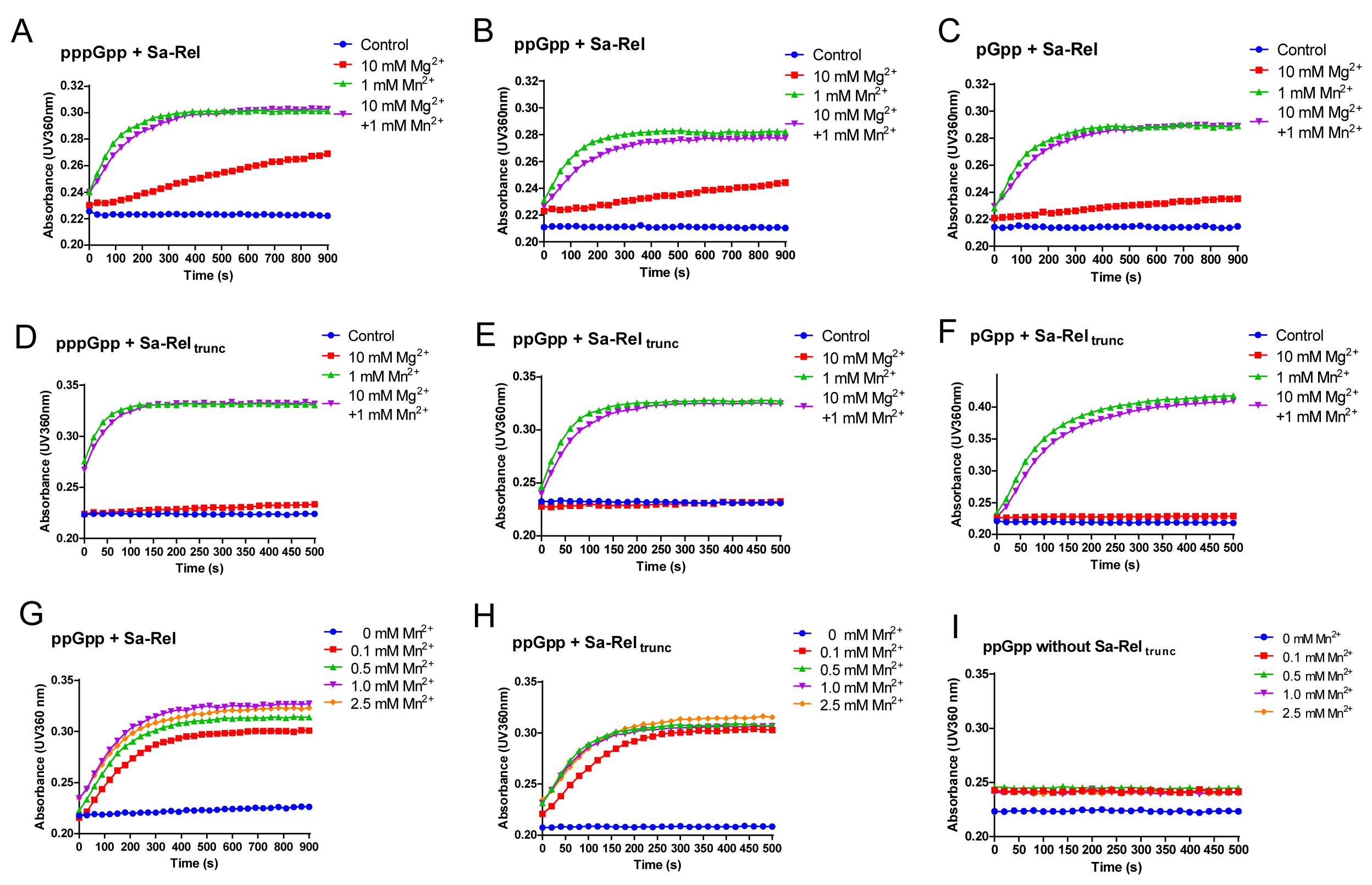

Supplement: S7 Fig — Panels A−C and D−F show the respective requirements for Mg2+/Mn2+ ions for (pp)pGpp hydrolysis by Sa-Rel and Sa-Reltrunc, under standardized conditions. Sa-Rel/Sa-Reltrunc mediated (pp)pGpp hydrolysis levels were quantified using enzyme-coupled continuous spectrophotometric phosphate-release assays over 0–500 s or 0–900 s (X-axis), with the UV absorbance at 360 nm (Y-axis) directly proportional to hydrolysis activity levels (equal to levels of pyrophosphate released). Representative data-sets are shown for each condition. Blue filled circles (control) no added metal ions; red filled squares: 10 mM Mg2+ added; green filled triangles: 1 mM Mn2+ added; purple filled inverted triangles: 10 mM Mg2+ + 1 mM Mn2+ added. Panels G and H show the effect of varying Mn2+ ion concentrations (0–2.5 mM) on the rate of ppGpp hydrolysis by Sa-Rel and Sa-Reltrunc, respectively. Blue filled circles: no Mn2+ ions added; red filled squares: 0.1 mM Mn2+ added; green filled triangles: 0.5 mM Mn2+ added; purple filled inverted triangles: 1.0 mM Mn2+ added; orange diamonds: 2.5 mM Mn2+ added. Panel I shows the results of control experiments performed in the absence of Sa-Rel/Sa-Reltrunc protein. See Materials and methods and S1 Appendix for experimental details. (TIF) [file pone.0213630.s009.tif]

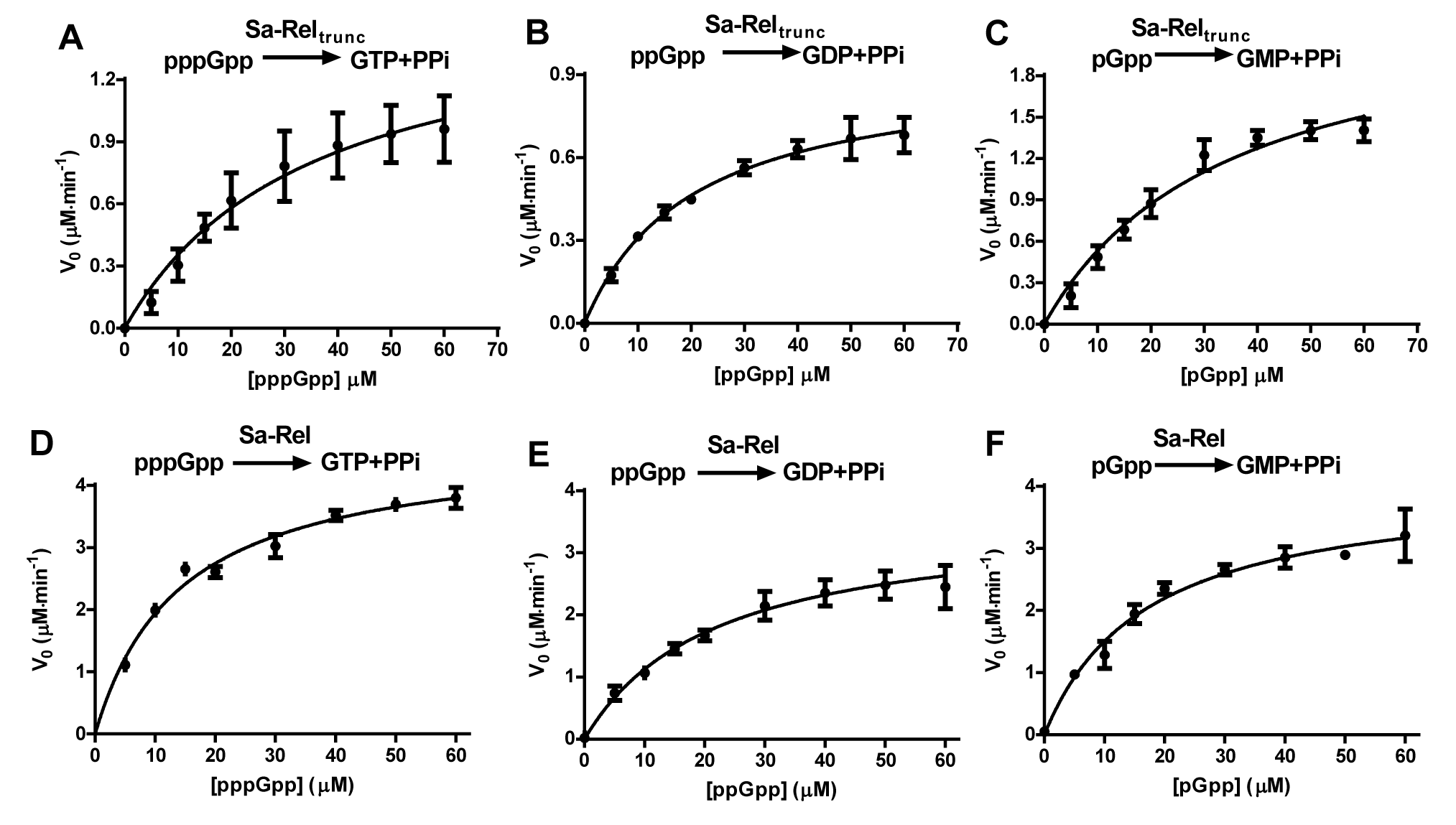

Supplement: S8 Fig — Panels A−C and D−F respectively show the plots of rate of (pp)pGpp hydrolysis by Sa-Rel and Sa-Reltrunc (V0; Y-axis, in units of μM / minute) versus (pp)pGpp substrate concentrations (X-axis; in micromolar units), for sets of assays performed to calculate the respective enzymatic kinetic parameters that are shown in Table 2. (A) pppGpp hydrolysis by Sa-Reltrunc; (B) ppGpp hydrolysis by Sa-Reltrunc; (C) pGpp hydrolysis by Sa-Reltrunc; (D) pppGpp hydrolysis by Sa-Rel; (E) ppGpp hydrolysis by Sa-Rel; (F) pGpp hydrolysis by Sa-Rel. Experimental details are described in the materials and methods section. 2–4 replicates were performed for each condition, with mean values plotted ± standard deviation. (TIF) [file pone.0213630.s010.tif]

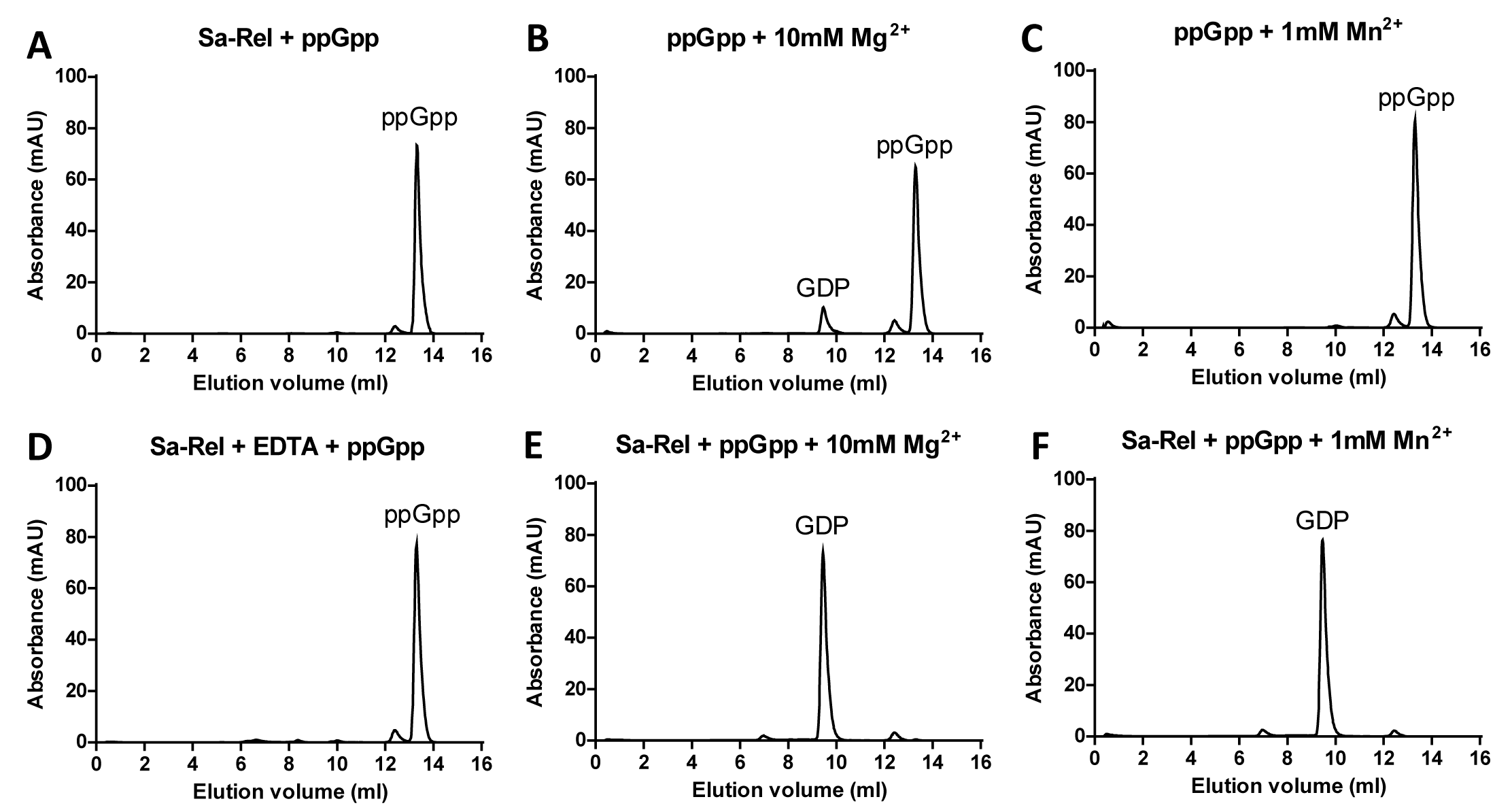

Supplement: S9 Fig — Panels A−F show representative anion-exchange chromatograms of product mixtures formed by the incubation of Sa-Rel with ppGpp in the presence/absence of Mg2+/Mn2+ ions or the divalent metal chelating agent EDTA, to determine which conditions enable hydrolytic activities. (A) Sa-Rel + ppGpp with no added metal or chelating agent; (B) ppGpp + 10 mM Mg2+ ions without Sa-Rel protein; (C) ppGpp + 1 mM Mn2+ ions without Sa-Rel protein; (D) Sa-Rel + ppGpp in the presence of 5 mM EDTA; (E) Sa-Rel + ppGpp + 10 mM Mg2+ ions; (F) Sa-Rel + ppGpp + 1 mM Mn2+ ions. The peaks corresponding to ppGpp or GDP (hydrolytic product) are respectively indicated on each chromatogram. All reactions were performed under standardized conditions, with minor modifications. Reaction mixtures (20 μl) contained 50 mM Tris-HCl (pH 7.8), 150 mM NaCl, 1 mM DTT, 1 mM MnCl2 or 10 mM MgCl2 or 5 mM EDTA, 0.5 mM ppGpp, 1 μM Sa-Rel protein, and were incubated at 25°C for 2h. In Panel D, Sa-Rel was pre-incubated with 5 mM EDTA for 10 min at 25°C immediately prior to its addition to the assay mixture. Product mixtures were analyzed by anion exchange chromatography, as described in the materials and methods section. (TIF) [file pone.0213630.s011.tif]

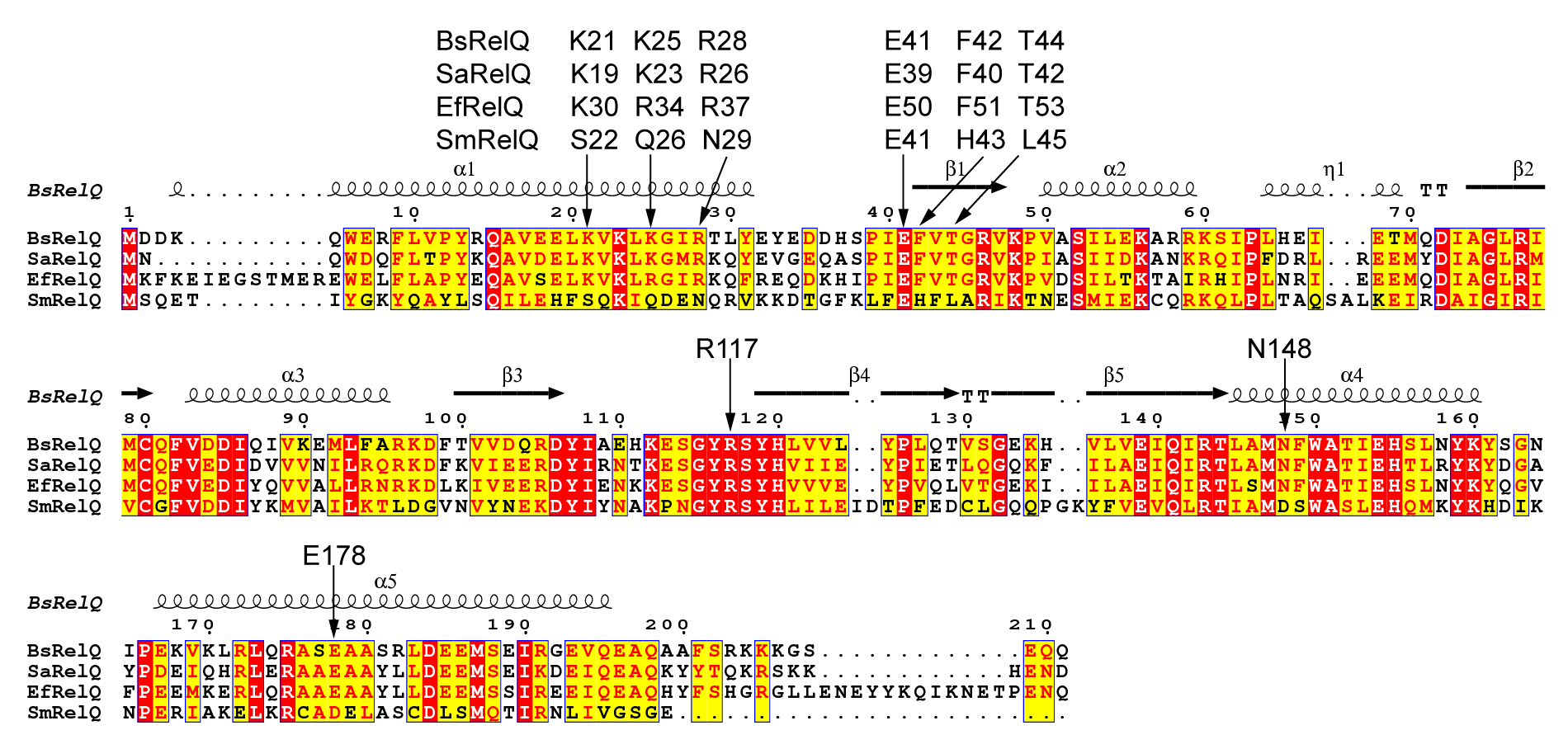

Supplement: S10 Fig — A multiple sequence alignment of the RelQ proteins from B. subtilis (BsRelQ), S. aureus (SaRelQ), E. faecalis (EFRelQ) and S. mutans (SmRelQ) is shown along with the corresponding secondary structure units determined for the BsRelQ protein [65]. Selected conserved residues implicated in substrate binding and allosteric regulation are identified for each RelQ protein. See S1 Appendix for further details. (TIF) [file pone.0213630.s012.tif]
