# Supplementary material for: The Ps and Qs of alarmone synthesis in Staphylococcus aureus
Source: PLoS One. 2019 Oct 15;14(10):e0213630. doi: 10.1371/journal.pone.0213630 (PMC6793942; doi:10.1371/journal.pone.0213630)
Supplement: S1 Appendix — (PDF) [file pone.0213630.s013.pdf]

The Ps and Qs of alarmone synthesis in *Staphylococcus aureus*

Ning Yang, Shujie Xie, Nga-Yeung Tang, Mei Y. Choi, Ying Wang, and Rory M. Watt

S1 Appendix. Supplementary Methods

Construction of Multiple Sequence Alignments of selected long-Rel proteins (S1 Fig) and RelQ proteins (S10 Fig)

Amino acid sequences were downloaded from the National Center for Biotechnology Information (NCBI) GenBank, and were aligned using CLUSTAL W, implemented in BioEdit v7.2.0. (Hall TA. BioEdit: a user-friendly biological sequence alignment editor and analysis program for Windows 95/98/NT. ***Nucl. Acids. Symp. Ser.*** 1999;41:95-98), formatted via T-Coffee [T-Coffee: A novel method for multiple sequence alignments Notredame, Higgins, Heringa, ***J Mol Biol.*** 2000;302:205-217. http://tcoffee.crg.cat/apps/tcoffee/index.html], exported to ESPritpt 3.0 (Robert X., Gouet P. Deciphering key features in protein structures with the new ENDscript server". ***Nucl Acids Res.*** 2014;42(W1):W320-W324. doi: 10.1093/nar/gku316. http://espript.ibcp.fr/ESPript/ESPript/) for visual rendering, using PDB 1VJ7 as a structural reference.

Qualitative analysis of inosine-based alarmone-like nucleotides synthesized by Sa-RelQ, Sa-RelP and EF-RelQ

Representative chromatograms are shown in **S2 Fig**.

All reactions were performed as described in the materials and methods section, except replacing GTP/GDP/GMP with ITP/IDP/IMP. Reactions (20 μl) contained 50 mM Bis-tris-propane pH 9.0, 150 mM NaCl, 1mM DTT, 1mM ATP, 1mM ITP/IDP/IMP, 2mM $MgCl_2$, 1 μM protein. The reaction mixtures were incubated 37°C for 2h, prior to analysis of product mixtures by anion exchange chromatography, as described in the materials and methods section.

Determination of optimum pH for ppGpp synthesis by Sa-RelQ, Sa-RelP and Sa-Rel$_{trunc}$

Plots are shown in **S3 Fig**.

All reactions were performed under standardized condition as described in the materials and methods section, with minor modifications. For Sa-RelQ and Sa-RelP, the rates of ppGpp synthesis were determined in reaction mixtures containing Bis-tris-propane buffer over the pH range pH 7.2–9.0. For Sa-Rel$_{trunc}$, the optimal pH was determined in Tris-HCl buffer over the pH range pH 6.8–8.8. The reaction mixtures were incubated 37°C for 2h, prior to analysis of product mixtures by anion exchange chromatography, as described in the materials and methods section. Reaction rates were calculated by quantifying AMP formation. 2–4 replicates were performed for each condition. Plots show the mean rate ± standard deviation.

Synthesis of (p)ppGpp by Sa-Rel and Sa-Rel$_{trunc}$

Representative chromatograms are shown in **S4 Fig**.

Reaction mixtures (20 μl) contained 50 mM Tris-HCl (pH 7.8), 150 mM NaCl, 1 mM DTT, 1 mM $MnCl_2$, 10 mM $MgCl_2$, 0.5 mM pGpp/ppGpp/pppGpp, 1 μM Sa-Rel or Sa-Rel$_{trunc}$ protein, and were incubated at 25°C for 2h. Product mixtures by anion exchange chromatography, as described in the materials and methods section.

Variation of (p)ppGpp-synthesis rates for Sa-Rel$_{trunc}$ with differing Mg^{2+} and nucleotide ratios.

Plots are shown in **S5 Fig**.

Assays were performed as described in the materials and methods section, with minor modifications. Specifically, the following Mg^{2+} ion concentrations (0.5, 2, 4, 6, or 10 mM) were used, in conjunction with various ratios of ATP and GTP/GDP (3:3, 1:3, 3:1, or 1:1 mM).

Reaction mixtures contained 1μM Sa-Rel_{trunc} protein in buffer containing 50 mM Tris-HCl (pH 7.8), 150 mM NaCl, 1 mM DTT, and were incubated at 25°C. Aliquots were removed for analysis at 5 different time points for analysis by anion exchange chromatography, as described in the materials and methods section. The following time points were used for reactions containing ATP + GTP: 1, 2, 4, 8, and 14 mins. The time points for ATP + GDP reaction mixtures were: 8, 14, 20, 30, 40 mins. 2–4 replicates were performed for each set of conditions, with error bars showing the mean rate ± standard deviation.

Hydrolysis of (pp)pGpp by Sa-Rel and Sa-Rel_{trunc}.

Representative chromatograms are shown in **S6 Fig**.

All reactions were performed under standardized conditions, as described in the materials and methods section, with minor modifications. Reaction mixtures (20 μl) contained 50 mM Tris-HCl (pH 7.8), 150 mM NaCl, 1 mM DTT, 1 mM $MnCl_2$, 10 mM $MgCl_2$, 0.5 mM pGpp/ppGpp/pppGpp, 1 μM Sa-Rel or Sa-Rel_{trunc} protein, and were incubated at 25°C for 2h. Product mixtures were analyzed by anion exchange chromatography, as described in the materials and methods section.

Metal ion requirements by Sa-Rel and Sa-Rel_{trunc} for (pp)pGpp hydrolysis activities.

Representative chromatograms are shown in **S7 Fig.**

The reactions were performed using the EnzChek phosphate assay kit, analogous to the procedure described in the materials and methods section (‘Quantification of alarmone hydrolysis by Sa-Rel and Sa-Rel_{trunc} proteins’), with minor modifications. The rates of P_i (phosphate) were determined in reaction buffer containing 10 mM $MgCl_2$ or (0, 0.1, 0.5, 1.0, 2.0 mM $MnCl_2$ (as indicated in the respective panels shown in **S6 Fig**). The following alarmone concentrations were used: pGpp (20 μM), ppGpp (15.66 μM), pppGpp (18.88 μM).
